# Spaceflight Virology: What Do We Know about Viral Threats in the Spaceflight Environment?

**DOI:** 10.1089/ast.2021.0009

**Published:** 2022-02-11

**Authors:** Bruno Pavletić, Katharina Runzheimer, Katharina Siems, Stella Koch, Marta Cortesão, Ana Ramos-Nascimento, Ralf Moeller

**Affiliations:** German Aerospace Center (DLR), Institute of Aerospace Medicine, Radiation Biology Department, Aerospace Microbiology Research Group, Linder Hoehe, Cologne (Köln), Germany.

**Keywords:** Virology, Space microbiology, Space medicine, Space travel, Decontamination, Virus diversity

## Abstract

Viruses constitute a significant part of the human microbiome, so wherever humans go, viruses are brought with them, even on space missions. In this mini review, we focus on the International Space Station (ISS) as the only current human habitat in space that has a diverse range of viral genera that infect microorganisms from bacteria to eukaryotes. Thus, we have reviewed the literature on the physical conditions of space habitats that have an impact on both virus transmissibility and interaction with their host, which include UV radiation, ionizing radiation, humidity, and microgravity. Also, we briefly comment on the practices used on space missions that reduce virus spread, that is, use of antimicrobial surfaces, spacecraft sterilization practices, and air filtration. Finally, we turn our attention to the health threats that viruses pose to space travel. Overall, even though efforts are taken to ensure safe conditions during human space travel, for example, preflight quarantines of astronauts, we reflect on the potential risks humans might be exposed to and how those risks might be aggravated in extraterrestrial habitats.

## 1. Introduction: Viruses in the Space Context

**FIG. 1.**
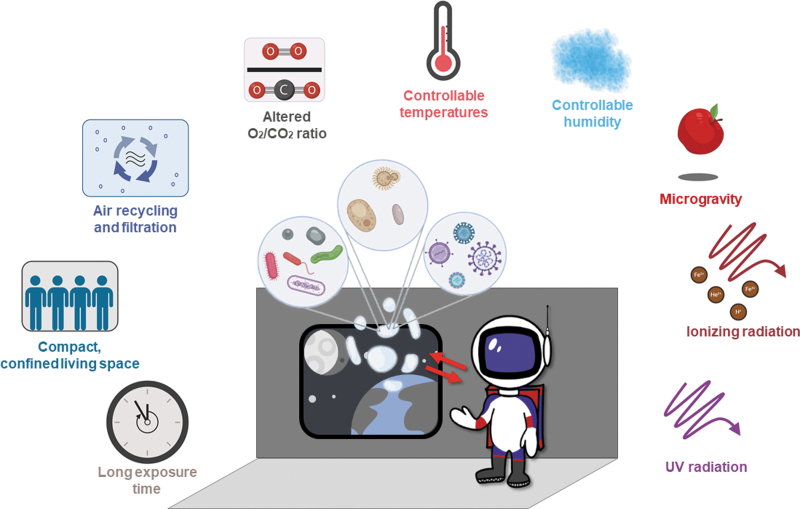
The environmental factors acting on microbes (including viruses), humans, and their interaction in space environments. Created with BioRender.com.

Space modules are enclosed, compact environments that harbor various microbial communities. Viruses are a significant part of such enclosed communities (Hjelmsø *et al.,* 2019; Mora *et al.,* 2019), some of which can be pathogenic to humans and pose a threat to individual and public health. However, viruses can also cause a range of other problems. For instance, bacteriophages can carry microbial virulence or antibiotic-resistance genes and spread them throughout bacterial populations on human bodies or in enclosed environments. Another example of the damage that viruses cause on Earth is the significant loss of crops grown for food, which is specific to plant viruses. Since human space missions are planned to mostly provide plant-based food to the astronauts, it is easy to see their threat to space travel. Therefore, plant and microbial viruses, along with human pathogenic viruses, represent a major issue for space travel. Space modules provide exceptional conditions for Earth's microbes to spread and grow (McKernan *et al.,* 2008) due to high radiation doses, microgravity, and compact spaces (Fig. 1).

Due to the extreme conditions in space, astronauts are especially vulnerable to infections given that cosmic radiation, microgravity, and psychological stress tend to compromise the human immune system (Crucian *et al.,* 2015; Fernandez-Gonzalo *et al.,* 2017; Akiyama *et al.,* 2020). Before departure to the International Space Station (ISS), crew members go through a 7-day isolation known as the “Health Stabilization Program” (NASA, 2010). Crucian and colleagues reported the occurrence of microbial diseases, cold sores, and allergies among 50% of the crew members in 38 six-month missions (Crucian *et al.,* 2016a).

Researching viruses is important in the space industry because the unique conditions of space travel can weaken human immunity. Therefore, viral infections during space travel may have a detrimental impact on the success of human missions. In contrast to the bacterial and fungal microbiomes, research on the virome variability in spacecraft is scarce (Berliner *et al.,* 2018). The study of viruses is challenging due to the need for a host, a low biomass in the environment, and a complex phylogeny. Nevertheless, new methods for high-throughput DNA-sequencing enable the collection of high amounts of environmental sequence data, which illustrates viral diversity (Roux *et al.,* 2017; Berliner *et al.,* 2018; Nooij *et al.,* 2018; Ann Gregory *et al.,* 2019). Metagenomics allows the detection of previously unknown viruses (Delwart, 2007) and has revealed that viruses constitute a major part of most microbiomes on Earth (Rosario and Breitbart, 2011; Mokili *et al.,* 2012). Additionally, other studies that have implemented both metagenomics and culture methods in buildings and transport vehicles have shown that most bacteria originated from the human skin both on surfaces and in the air of enclosed spaces (Tsai and Macher, 2005; Gibbons *et al.,* 2015; Hsu *et al.,* 2016; Stephens, 2016). Yet it is not clear whether the same assumption holds for viruses. Literature reports on viral diversity in closed environments vary, depending on the source of microbes (Prussin and Marr, 2015; Prussin *et al.,* 2019, 2020). Prussin and Marr identified the outside environment as the major source of microbes in an indoor environment (Prussin and Marr, 2015). Also, metagenomic studies on the seasonality of microbial distribution in bioaerosols suggest that humans in the enclosed environments strongly affect the airborne viral communities (Prussin *et al.,* 2019). On a space station, however, the source of microbes is represented by the interchange of astronaut and microbe cross-contamination from humans to equipment and subsequently from equipment to humans. The low numbers of passengers make the ISS a microbiologically controlled environment. Additionally, it allows for thorough microbial monitoring of equipment or the astronauts themselves by the implementation of strict hygiene measures, methods of sterilization, and food monitoring (Pierson *et al.,* 2013). Nevertheless, the ISS might have a very dynamic virome.

To better understand how we can monitor and control viral spread in space travel, the present study addresses four main questions:

- What is the abundance and diversity of viruses in the ISS microbiome?
- How are viruses and their human hosts influenced by the environmental conditions of space travel?
- How can viruses be monitored and, in the case of harmful contaminations, decontaminated during space missions?
- Are there any health threats associated with viruses in the space context?

## 2. Viruses in the Microbiome of the ISS Surfaces

**FIG. 2.**
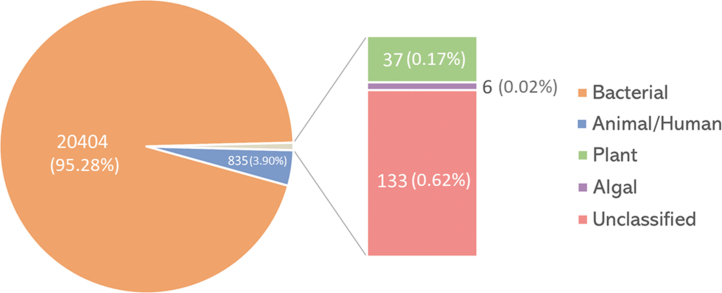
Distribution of viruses by the number of reads detected on ISS surfaces. The total number of detected reads is designated for every category. Data from the work of Mora *et al.* (2019). Created with MS Excel v2102.

**FIG. 3.**
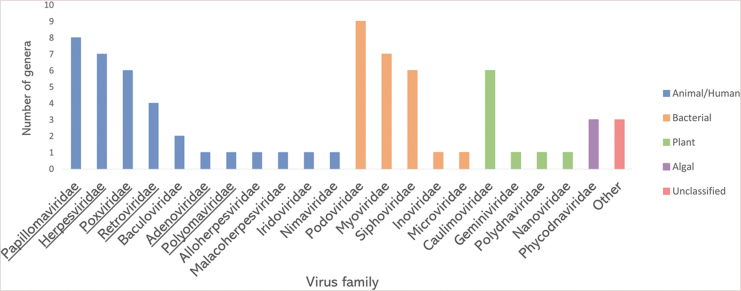
Virus families detected on ISS surfaces by shotgun metagenomic analysis. For every family, the number of detected genera is plotted. The families that contain human pathogens are underlined. Data from the work of Mora *et al.* (2019). Created with MS Excel v2102.

Our understanding of the viral microbiome dynamics on the ISS is sparse, mostly due to limited methodologies with which to study it. So far, there has only been one analysis of the ISS microbiome that included viruses (Mora *et al.,* 2019). Therein, shotgun metagenomic sequencing of environmental surface swabs characterized the microbiome inside the ISS. The sequenced reads were then assigned based on sequence similarity to phylogenetic groups in virus genome databases. The reads similar to virus sequences made up 0.57% (21,415 out of 3,731,403) of all sequence reads. The majority of virus-related reads (∼95%) originated from bacteriophages, while ∼4% were derived from animal/human viruses, including herpesviruses, and the remaining were classified as reads related to plant and algal viruses or remained unclassified (Fig. 2). Among them, the reads similar to viruses from 72 different virus genera were identified to be distributed in 21 families, including the ones that contain human pathogens (Fig. 3). It is also worth noting that the metagenomic analysis was performed only on the pooled subset of environmental samples. The average length of reads was 126 bp. Those are relatively short reads. Therefore, some viruses might have been missed during the analysis.

The low abundance of reads similar to virus sequences may be due to the highly sterile conditions on the ISS or caused by the decreased stability of virus samples in comparison to other microbes. Also, the viral genomes are underrepresented in genomic databases used for assigning sequences, so a great portion may remain unidentified. Bacteriophages influence the human microbiome and physiology by altering an organism's microbiome (Navarro and Muniesa, 2017) with potential impacts on the astronauts' health. Reads similar to animal viruses were distributed into 33 genera, 13 of which are known to infect humans and cause diseases of varying severity. They include a range of herpesviruses, which establish latency and can undergo reactivation (Pierson *et al.,* 2005; Mehta *et al.,* 2014, 2017; Rooney *et al.,* 2019; Voorhies *et al.,* 2019). These authors' analysis results indicate that pathogenic viruses were present in low abundance and unlikely to cause significant health problems on short-term space missions, even under conditions unfavorable to a healthy immune system. However, their impact on long-term missions remains unknown.

## 3. The Influence of Environmental Factors Related to Space Travel on Viruses and Their Hosts

During space travel, humans and their microbiome are exposed to conditions that significantly differ from those in their natural environment. As the ISS orbits Earth at around 420 km above sea level, exposure to cosmic and UV radiation is much higher than on the ground due to the filtration of UV by the ozone layer. There is also the additional stress of microgravity in space. Furthermore, space missions can last 3–6 months, and future missions could last up to a few years. These environmental factors can affect virus integrity directly and influence their stability or indirectly influence the host vulnerability to infection (Foster *et al.,* 2014; Carratalà *et al.,* 2017).

As human space missions are planned more robustly than ever, it is also necessary to consider the effects of the space environment on viral infectivity and environmental stability. This is a highly complex topic given that viruses are influenced by a range of environmental factors related to space travel, depending on the mission. Some examples include extreme and rapid temperature variations during day/night cycles on the Moon, extremely low pressures of the Moon and Mars, or microgravity in deep space. Also, the fine regolith dust of varying chemical composition, present on many rocky celestial bodies, can potentially affect the stability of a viral particle. Since astronauts will spend a long time confined in enclosed habitats, as they currently do on the ISS, the conditions inside them would be the most relevant for human health and virus spread. Therefore, for this mini review, we summarized the effects of physical conditions that affect viruses and their hosts in space habitats, that is, elevated levels of UV and ionizing radiation, humidity since it is an important factor of the enclosed environment that affects viral spread, and microgravity because it is currently impossible to control and has a considerable effect on virus-host interaction. A summary of studies addressing the impact of space-related stresses on virus stability is presented in Table 1. Also, the physiological stresses for humans in such isolated conditions include psychological stress, nutrient availability, close contact with other crew members, artificial light/dark cycles inside the habitat, among others. Even though these are important factors to consider in the future, they are not covered here as their consequences vary among individuals and bear a minimal source of concern in comparison to the physical factors explored in this review.

**Table 1.** Summary of the Studies of Individual Simulated Stress Factors Acting on Viruses during Air and Space Travel

Environmental factor	Effect on viruses/host	Tested viruses	Host	Transmission	References
UV radiation	reactivation in host	Human papillomavirus	Human	Mucosal contact	Viarisio *et al.,* 2011
		Rat cytomegalovirus	Rat	Blood, saliva, transplacental	Garssen *et al.,* 1995
		Murine herpes simplex virus 1	Mouse	Mucosal contact, saliva	El-Ghorr and Norval, 1996; Goade *et al.,* 2001
	genome damage	Poliovirus	Human	Ingestion of food/water, inhalation of aerosols	Simonet and Gantzer, 2006
		Herpes simplex virus	Human	Mucosal contact, saliva	Mirshafiee *et al.,* 2012
		Mengovirus	Mouse	Inhalation of aerosols	Miller and Plagemann, 1974
		Murine polyoma virus	Mouse	Inhalation of aerosols	Lytle and Sagripanti, 2005; Huang *et al.,* 2016
		Encephalomyocarditis virus	Rodents, pig	Ingestion of food/water	Lytle and Sagripanti, 2005
		Adenovirus	Mammals	Contact-based, inhalation of aerosols	Eischeid and Linden, 2011
		Vesicular stomatitis virus	Livestock	Contact-based, ingestion of food/water, inhalation of aerosols	Mirshafiee *et al.,* 2012
		Cowpea mosaic virus	Cowpea plant	Insects, sap inoculation	Rae *et al.,* 2008
		Bacteriophage T7	*E. coli*	Contact-based	Fekete *et al.,* 2008
		Bacteriophage GA	*E. coli*	Contact-based	Simonet and Gantzer, 2006
		Bacteriophage MS2	*E. coli*	Contact-based, aerosols	Lytle and Sagripanti, 2005; Simonet and Gantzer, 2006
		Bacteriophage Qbeta	*E. coli*	Contact-based, aerosols	Lytle and Sagripanti, 2005; Simonet and Gantzer, 2006
		Bacteriophage F2	*E. coli*	Contact-based, aerosols	Lytle and Sagripanti, 2005
	viral surface damage	Reovirus	Human	Ingestion of food/water	Subasinghe and Loh, 1972
		Mengovirus	Mouse	Inhalation of aerosols	Miller and Plagemann, 1974
		Adenovirus	Mammals	Contact-based, inhalation of aerosols	Eischeid and Linden, 2011
		Bacteriophage MS2	*E. coli*	Contact-based	Wigginton *et al.,* 2010, 2012
Ionizing radiation	increased illness severity	Herpes simplex virus	Human	Mucosal contact, saliva	Openshaw *et al.,* 1979
		Murine herpes simplex virus 1	Mouse	Mucosal contact, saliva	Wang *et al.,* 1990
		Theiler's murine encephalitis virus	Mouse	Contact-based, ingestion of food/water	Rodiriguez *et al.,* 1990
		Rabies virus	Mouse	Contact-based, saliva	Ceccaldi *et al.,* 1996
		Bacterial prophage	*E. coli*	Contact-based	Parfenov and Lukin, 1973
	genome damage	Poliovirus	Human	Ingestion of food/water, inhalation of aerosols	Ward, 1980
		Murine norovirus 1	Mouse	Ingestion of food/water, inhalation of aerosols	Feng *et al.,* 2011
		Porcine parvovirus	Pig	Contact-based, ingestion of food/water	Summers and Szybalski, 1967; Ward, 1980; Grieb *et al.,* 2002; Feng *et al.,* 2011
		Vesicular stomatitis virus	Livestock	Contact-based, inhalation of aerosols, insects	Feng *et al.,* 2011
		Bacteriophage phi 29	*B. subtilis*	Contact-based	Summers and Szybalski, 1967
	viral surface damage	Poliovirus	Human	Ingestion of food/water, inhalation of aerosols	Ward, 1980
		Human adenovirus	Human	Ingestion of food/water, inhalation of aerosols	Pimenta *et al.,* 2016
		Murine norovirus 1	Mouse	Ingestion of food/water, inhalation of aerosols	Feng *et al.,* 2011
		Vesicular stomatitis virus	Livestock	Contact-based, ingestion of food/water, inhalation of aerosols, insects	Feng *et al.,* 2011
		Tobacco mosaic virus	Tobacco plant	Direct contact between leaves	Koike *et al.,* 1992
Humidity (high)	reduces survivability or transmission of enveloped viruses	Influenza	Human	Contact-based, inhalation of aerosols	Harper, 1961; Schaffer *et al.,* 1976; Noti *et al.,* 2013; Marr *et al.,* 2019
		Severe acute respiratory syndrome coronavirus-1	Human	Inhalation of aerosols	Chan *et al.,* 2011
		Severe acute respiratory syndrome coronavirus-2	Human	Inhalation of aerosols	Wang *et al.,* 2020; Haque and Rahman, 2020
		Human coronavirus-229E	Human	Inhalation of aerosols	Ijaz *et al.,* 1985
		Langat virus	Human	Insects	Benbough, 1971
		Respiratory syncytial virus	Human	Contact-based, inhalation of aerosols	Tang, 2009
		Parainfluenza viruses	Human	Contact-based, inhalation of aerosols	Tang, 2009
		Measles virus	Human	Contact-based, inhalation of aerosols	Tang, 2009
		Rubella virus	Human	Inhalation of aerosols, transplacental	Tang, 2009
		Varicella zoster virus	Human	Contact-based	Tang, 2009
		Semliki forest virus	Mammals and birds	Inhalation of aerosols, insects	Benbough, 1971
		Venezuelan equine encephalomyelitis	Horse	Insects	Harper, 1961
		Vesicular stomatitis virus	Livestock	Contact-based, inhalation of aerosols, vector-based	Songer, 1967
		Rous sarcoma virus	Chicken	Contact-based	Webb *et al.,* 1963
		Newcastle disease virus	Birds	Contact-based, ingestion of food/water, inhalation of aerosols	Songer, 1967
Humidity (low)	reduces survivability of non-enveloped viruses	Polio virus	Human	Ingestion of food/water, inhalation of aerosols	Harper, 1961
		Rhinovirus-14	Human	Contact-based, inhalation of aerosols	Karim *et al.,* 1985
		Vesicular exanthema virus	Pig	Ingestion of food/water, inhalation of aerosols	Donaldson and Ferris, 1976
		Feline calicivirus	Cat	Contact-based of infected mucosa, saliva	Donaldson and Ferris, 1976
		Bacteriophage T7	*E. coli*	Ingestion	Benbough, 1971
	improved inactivation with UV radiation	Bacteriophage MS2	*E. coli*	Ingestion	Tseng and Li, 2005
		Bacteriophage phi X174	*E. coli*	Ingestion	Tseng and Li, 2005
		Bacteriophage phi 6	*Pseudomonas* bacteria	Contact-based	Tseng and Li, 2005
		Bacteriophage T7	*E. coli*	Ingestion	Tseng and Li, 2005
Microgravity	*in vitro* inhibits reactivation in host cells	Kaposi's sarcoma-associated herpesvirus	Human	Blood, ingestion of food/water, mucosal contact	Honda *et al.,* 2020
	increased illness severity	Herpes simplex virus	Human	Contact-based of infected mucosa, saliva	Fuse and Sato, 2004
	may promote viral spread across an organism	Indirect evidence on FITC-dextran particles	—	—	Alvarez *et al.,* 2019

The routes of infection and host for each virus are specified. Herein are included bacteriophages due to the direct impact in bacterial symbionts affecting the host microbiome.

**FIG. 4.**
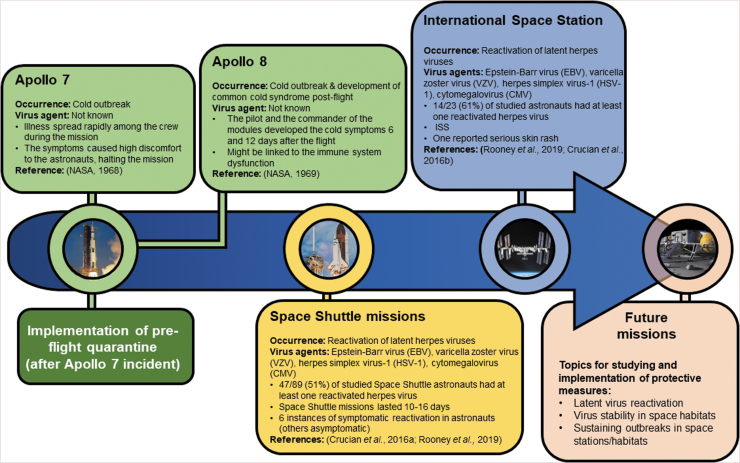
The occurrence of reported virus-related events over spaceflight history. The 7-day preflight quarantine of the astronauts helped sustain the infectious diseases on space missions as there are no reported outbreaks after its implementation. However, this might also be due to confidentiality of astronaut medical data. Currently, only reactivation of herpes viruses is being reported as a problem on space missions; though this is generally asymptomatic, it causes health issues, especially in the long term.

Over the course of spaceflight history, there has only been one reported outbreak in space—the common cold (head cold) outbreak among the three Apollo 7 astronauts, which spread rapidly and reduced the ability of the astronauts to cooperate with the control center (NASA, 1968). The reasons for the lack of reports are mainly pre/flight quarantine, normal mission duration of up to 6 months, but also the confidentiality of the astronaut medical data (Crucian *et al.,* 2016a). However, reactivation of latent viruses has been documented in astronauts on the ISS, which caused skin rash and rhinitis in some exceptional cases (Crucian *et al.,* 2016b). Varicella zoster virus (VZV) is one of the viruses reactivated in astronauts, which is known to cause significant pain and tissue damage in some cases. Therefore, vaccination of astronauts against VZV will be helpful to reduce the symptoms during space missions. Figure 4 illustrates the reported virus-related events over spaceflight history.

### 3.1. UV radiation

Most damaging UV radiation is filtered by Earth's atmosphere; therefore, all species on Earth are protected from most of the UVC, some UVB, and UVA to a lesser extent (De Gruijl and Van der Leun, 2000). UV radiation is one of the most threatening and damaging factors in the space environment for humans and microorganisms, along with ionizing radiation (gamma rays, X-rays, and fast charged particles). Those factors are also of concern on the Moon and Mars (Furukawa *et al.,* 2020). UV has a major impact on viruses as discussed below. Interestingly, it has been proposed that viral populations can contain subgroups that are more resistant to UV inactivation as explained by the two-hit model of inactivation, which postulates that “two hits” of radiation rays are required to inactivate a virus particle (Kowalski *et al.,* 2000; Cutler *et al.,* 2011).

Ultraviolet radiation substantially reduces virus titers on surfaces and in the air (Tseng and Li, 2005, 2007; Sagripanti and Lytle, 2011). UVC radiation at 254 nm wavelength can induce damage to the viral genome and proteins (Beck *et al.,* 2014, 2016, 2018). Overall, DNA viruses tend to be more resistant than RNA viruses, and those with double-stranded genomes are more resistant to UV radiation (Tseng and Li, 2007). Yet, independently of the nucleic acid type, genome damage is the most important factor for viral inactivation (Wang *et al.,* 2004; Ye *et al.,* 2018).

Also, UV radiation can reactivate dormant viruses in rodents (Garssen *et al.,* 1995; El-Ghorr and Norval, 1996; Norval and El-Ghorr, 1996; Goade *et al.,* 2001; Norval, 2006; Viarisio *et al.,* 2011). Epidemiological data on papillomaviruses and herpesviruses suggest the same assumption holds for humans (Chen *et al.,* 2008; Hampras *et al.,* 2014; Uberoi and Lambert, 2017). The mechanism of this reactivation is related to how UV radiation suppresses the immune system (Norval and Halliday, 2011; Schwarz and Schwarz, 2011; Ullrich and Byrne, 2012) by activation of regulatory T cells (thymus cells, a type of lymphocyte) that produce immunosuppressive signals and thus inhibit the immune system (Rana *et al.,* 2008). This paves the way for opportunistic disease-causing viruses to exploit a compromised immune system and provoke disease. Therefore, treatments that influence T cell signaling might impact the astronaut's health and contribute to virus control.

### 3.2. Ionizing radiation—gamma rays, X-rays, and charged particles

Ionizing radiation is another factor of space travel that influences living organisms, which are shielded from its effects on Earth due to the planet's magnetic field and the ozone layer. Those are gamma and X-rays that originate from the Sun or charged ions (H^+^, He^2+^, Fe ions) coming from interstellar space (Horneck *et al.,* 2010; Chancellor *et al.,* 2018). This type of radiation can induce significant damage to biomolecules by causing the formation of reactive oxygen species (ROS) and by breaking the molecular bonds (Reisz *et al.,* 2014; Cortesão *et al.,* 2020) and cause double-stranded breaks in DNA (Vignard *et al.,* 2013).

Long-term exposure to low-intensity ionizing radiation increases the chances of developing cancer, especially of the skin and lungs (Cohen, 2002; WHO, 2016). Rodent models show that both gamma and X-rays have immunosuppressive effects that lead to the reactivation of herpes and rabies viruses and an increase in illness severity (Openshaw *et al.,* 1979; Rodiriguez *et al.,* 1990; Wang *et al.,* 1990; Ceccaldi *et al.,* 1996). One of the most dramatic effects is the apoptosis of dendritic cells, which neutralizes their defensive action that controls B and T cell immune response (Liu *et al.,* 2011).

Ionizing photons also inactivate environmental viruses directly and indirectly (Johnson, 1965; Sullivan *et al.,* 1971; Ward, 1980; Hume *et al.,* 2016) mainly by damaging viral genomes and thus compromising viral replication (Summers and Szybalski, 1967; Ohshima *et al.,* 1996; Lomax *et al.,* 2013). Direct inactivation acts by damaging the viral genome, proteins, and lipids. The indirect mechanism of inactivation acts through the generation of hydroxyl radicals from water, ozone, and oxygen molecules, which originates ROS and which then damages nucleic acids, proteins, and lipids.

Studies suggest that different types of radiation can induce the lytic cycle of herpesviruses—Epstein–Barr virus (EBV) and human herpesviruses (HHV) types 1–3 (Ramirez-Fort *et al.,* 2018; Mehta *et al.,* 2018). Hence, while immunosuppression plays a role in the reactivation of herpesviruses, ionizing radiation can also directly activate lysogenic viruses (Ramirez-Fort *et al.,* 2018).

### 3.3. Humidity

Humidity is another important environmental factor that can be controlled in space modules and has a known impact on viruses and other microbes (Yamaguchi *et al.,* 2014). It also influences the virus-host interaction. Some studies have shown that relative humidity (RH) can be a predictor of viral stability (Shaman and Kohn, 2009; Tang, 2009). In these studies, stability depended on the presence of a lipid envelope. Viruses with an envelope conveyed more stability at lower RH (20–30%), while those without an envelope and a protein shell are more stable at higher RH (70–90%). However, recent studies on influenza and SARS-CoV-2 suggest that absolute humidity (AH) is a better predictor for stability (Deyle *et al.,* 2016; Marr *et al.,* 2019; Haque and Rahman, 2020; Wang *et al.,* 2020). At higher AH, respiratory viruses show reduced transmission. This is because high AH can be only reached at higher temperatures, so the effect of AH is the combined action of RH and high temperature (Marr *et al.,* 2019). In addition to inactivating airborne infectious viruses, high humidity thickens human mucosa, which acts as the barrier between the organism and environment, therefore reducing the possibility of viral infection from the air (Kudo *et al.,* 2019). In the case of viral outbreaks in space habitats, humidity could be regulated to decelerate viral spread.

Hypothetically, viruses with lipid envelopes accumulate on the surface of water droplets in the air and are afterward inactivated by surface tension (Yang and Marr, 2012). At increased RH, droplets are larger because there is less evaporation that leads to a higher accumulation of lipid-enveloped viruses being inactivated by surface tension. On the other hand, non-enveloped viruses are repelled by water surfaces, which makes inactivation less efficient. Also, higher evaporation rates at lower RH decrease the pH of aerosol droplets, which induces conformational changes on surface proteins. This can make non-enveloped viruses less stable (Yang and Marr, 2012). However, these scenarios are currently only hypotheses, and more mechanistic research is needed to understand virus inactivation by RH and AH. Accordingly, bacteriophages with a protein envelope are better inactivated by UV radiation when exposed to low RH (Tseng and Li, 2005, 2007), but for a porcine reproductive and respiratory syndrome virus (PRRSV), a moderate RH (25–79%) enhances UV inactivation (Cutler *et al.,* 2012) for unknown reasons.

### 3.4. Microgravity

In space travel, exposure to varying *g*-forces is very common. Spacecraft experience high *g*-forces during liftoff and are under the influence of microgravity once they reach orbit. Also, future celestial destinations like the Moon and Mars have lower gravitational force than Earth. Therefore, research of viruses in microgravity-like conditions is more relevant in the context of space travel, as well as hypergravity, which is relevant in the consideration of high *g*-forces experienced during launching into orbit and orbital transfers.

Research on virus-host interactions in microgravity scenarios indicates that it results in beneficial effects, contrary to other factors that induce viral reactivation (*e.g.,* ionizing radiation), (Honda *et al.,* 2020). Honda and colleagues found that cells infected with Kaposi's sarcoma-associated herpesvirus (KSHV) upregulate cell-intrinsic KSHV-inhibiting restriction factors in microgravity like CCCTC-binding factor (CTCF) or adenosine monophosphate–activated protein kinase (AMPK). This suggests that microgravity alone could partially counteract the damaging or debilitating effects of other space travel stress factors.

However, additional evidence displays a negative impact of microgravity. The intestinal epithelial cells have been shown to experience a decrease in their epithelial barrier function under microgravity (Alvarez *et al.,* 2019). The gut harbors its microbiome, and it prevents viruses and other microbes from entering inside an organism. In this research, ion flux and fluorescein isothiocyanate-dextran (FITC-dextran) permeability of human epithelial barrier were measured *in vitro,* and it was found that the permeability increased, potentially allowing viral spread.

## 4. Control of Viruses in Space Travel

Innovative and efficient ways for virus identification, tracking, and inactivation are crucial to tackling the vast spectrum of problems they cause in public health, the economy, and agriculture. This is also important in spaceflight to prevent hindering missions due to viral infections. Various measures are taken to ensure the sanitary conditions aboard spacecraft, from microbial tracking to preflight astronaut isolation (Pierson *et al.,* 2013).

Additional decontamination is applied to spacecraft with a special focus on bacterial and fungal spores, preventing microbial spread to other celestial bodies. Developing novel methods of disinfection and microbial control in space travel poses a challenge because they should efficiently fulfill the healthcare requirements compatible with human exposure without affecting the structural integrity of a spacecraft. For this reason, chemical disinfection is often not suitable for space applications.

### 4.1. Antimicrobial surfaces

Certain materials and compounds have antimicrobial properties that are exploited to inhibit or reduce microbial growth in environments where strict hygiene standards are necessary, such as airplanes, spaceflight, healthcare, or food production (Page *et al.,* 2009; Mousavi Khaneghah *et al.,* 2018).

Metals with antimicrobial properties are copper, silver, and their respective alloys, but also ions of mercury, iron, lead, zinc, and aluminum (Sreekumari, *et al.,* 2005). Including a minimum of 55% of copper into composite materials (Mehtar *et al.,* 2008) would contribute to ensuring safety during air and space travel as it efficaciously inactivates most viruses, including SARS-CoV-2 and other microbes (Noyce *et al.,* 2007; Warnes *et al.,* 2015; Schmidt *et al.,* 2017; Bryant *et al.,* 2021). There are also efforts to develop alternative antimicrobial surfaces for space travel such as AGXX (Van Loi *et al.,* 2018), a silver/ruthenium surface coating that produces ROS, which inactivates most pathogens, including viruses.

### 4.2. Sterilization of spacecraft

There is a concern to protect other planets from contamination with Earth's microbes. Spacecraft surfaces are sterilized inside and out by intense treatment at high temperatures (145°C) for several days, which guarantees that no microbes or spores can survive. In recent years, plasma sterilization has been considered an effective alternative due to its more convenient application (Stapelmann *et al.,* 2013). These methods of sterilization are collaterally highly effective against viruses (Bozkurt *et al.,* 2015; Sakudo *et al.,* 2019), making antimicrobial disinfection overly effective in preventing virus contaminations.

Interestingly, full microbial sterility of spacecraft in human missions is hard, if not impossible, to achieve because astronauts themselves represent a reservoir of microbes that can cross-contaminate the environment. Development of simple and easy-to-use detection tests such as SHERLOCK (Gootenberg *et al.,* 2018) or DETECTR (Chen *et al.,* 2018) for specific viral genomes would be helpful in space missions. Such tests utilize CRISPR-Cas9 technology to specifically detect viral sequences within minutes. Also, environmental nucleic acid sequencing techniques like Oxford Nanopore (Quick, 2019) are becoming more robust and easier for application in extreme environments such as space missions. This will vastly aid the detection and characterization of viromes in enclosed environments, not just in space habitats, but also on Earth.

### 4.3. Air filtration

Inside the ISS, there is constant air circulation and filtration with high-efficiency particulate air (HEPA) filters. They have been reported to efficiently filter out small aerosols (98% efficiency, particles 0.3–10 μm diameter; Mousavi *et al.,* 2020). Even though virus sizes are in nanometer range, they travel in air-suspended droplets and aerosols that are micrometer-sized, being efficiently filtered by HEPA filters. Therefore, they protect the astronauts. Those filters also provide an interesting test sample for researching airborne microbial communities on the ISS. Probably, future space habitats will also include such filters due to the need for constant air recycling.

## 5. Health Threats Viruses Pose to Space Travel

### 5.1. Latent infections and viral reactivation

Even though disinfection on space missions is thorough, it is impossible to completely neutralize the disease-inducing factors due to their prevalence in the human hosts, since a large portion of humanity is already latently infected with specific viruses. Latent infections are caused by viruses that, upon a single inoculation, can establish lifelong infections, like herpesviruses. Table 2 lists herpesviruses that cause latent infection in humans regarding global prevalence, route of transmission, possible consequences, and site of persistence. As a result of immune deregulation in space, these viruses can undergo reactivation, potentially with increased severity due to the vulnerability of the host. Their reactivation in astronauts is probably caused by the stress factors discussed above, resulting in the changes in CD8+ T cell (T lymphocytes expressing *cluster of differentiation 8* glycoprotein) and regulatory T cell function, which have been described to maintain viral latency (Mehta *et al.,* 2013).

**Table 2.** The Global Prevalence, Route of Transmission, Some Possible Consequences of Primary Infection and Reactivation, and Site of Persistence of Herpesviruses Causing Latent Infections in Humans: HSV-1 and HSV-2, VZV, EBV, HCMV, HHV-6 and HHV-7, and KSHV

Viruses	Global prevalence	Transmission	Possible consequences	Site of persistence	References
HSV-1	67% (age under 50)	Contact-based of infected mucosa	Cold sores, genital ulcers, related skin lesions, keratitis, encephalitis, meningitis	Sensory and cranial nerve ganglia	Grinde, 2013
HSV-2	11.3% (age 15–49) the highest burden in Africa	Contact-based of infected mucosa	Cold sores, genital ulcers, keratitis, encephalitis, meningitis, Mollaret's meningitis	Sensory and cranial nerve ganglia	Grinde, 2013; Looker *et al.,* 2015
VZV	>90% (before adolescence, pre-vaccination era, high-income countries)	Contact-based, inhalation of aerosols	Chickenpox, herpes zoster	Sensory and cranial nerve ganglia, spinal cord	Grinde, 2013; WHO, 2014
EBV	<90% (lifetime)	Blood, contact-based of infected mucosa, saliva	Hairy leukoplakia, periodontitis, nasopharyngeal carcinoma, mononucleosis, lymphoma, Hodgkin's lymphoma	Memory B cells	Chang *et al.,* 2009; Grinde, 2013; Ozturk *et al.,* 2020
HCMV	83%	Blood, mucosal contact during breastfeeding, saliva, urine	Mononucleosis, colitis, esophagitis, retinitis, pneumonia, hepatitis, and encephalitis	Monocytes, lymphocytes, and epithelia	Grinde, 2013; Zuhair *et al.,* 2019; Sezgin *et al.,* 2019
HHV-6	70–100%	Contact-based of infected mucosa, saliva	Exanthema subitum, encephalitis, fulminant hepatitis, liver dysfunction, thrombocytopenia, hemophagocytic syndrome	Various leukocytes	De Bolle *et al.,* 2005; Grinde, 2013
HHV-7	75–98% except Northern Japan: 44%	Contact-based, saliva	Exanthema subitum, encephalitis	T cells, epithelia	Krueger *et al.,* 1998; Ward, 2005; Grinde, 2013
KSHV	Geographic differences: >1.5% (adults in USA); 55% (Uganda)	Blood, contact-based of infected mucosa, saliva	Kaposi's sarcoma, Castleman disease	B cells	Engels *et al.,* 2007; Biryahwaho *et al.,* 2010; Grinde, 2013; Fajgenbaum and Shilling, 2018

B cells = bursa cells, a type of lymphocyte; EBV = Epstein–Barr virus; HCMV = human cytomegalovirus; HHV = human herpesvirus; HSV = herpes simplex virus; KSHV = Kaposi's sarcoma-associated herpesvirus; T cells = thymus cells, a type of lymphocyte; VZV = varicella zoster virus.

Multiple studies have detected reactivation and shedding of viruses in human space and analog missions and environments (Pierson *et al.,* 2005, 2007; Mehta and Pierson, 2007; Mehta *et al.,* 2014, 2017). Due to the prevalence of herpesviruses in the general population, reactivation events cannot be reliably avoided in space either by isolation or by medical treatment. Therefore, developing spaceflight countermeasures to attenuate viral reactivation outcomes such as preflight immunity enhancement to inhibit viruses is a factor to be considered. Though herpesviruses are not the only viruses that latently infect humans, they are the major focus in spaceflight (Rooney *et al.,* 2019). Thus, further studies of latent viral infections are necessary to determine the vulnerability of astronauts to other latent infection viruses besides those belonging to the Herpesviridae family.

The reactivation of these viruses, some of which are associated with increased mortality (Ren *et al.,* 2020), has also been detected in astronauts. Besides the cold-sore-causing HSV-1 (Crucian *et al.,* 2016a), the reactivation of other herpesviruses such as EBV (Payne *et al.,* 1999), human cytomegalovirus (HCMV; Vuong *et al.,* 2000), and VZV has been observed in astronauts before with mild symptoms (Mehta *et al.,* 2004). Though serious consequences have not yet been observed in astronauts, this might be due to the currently short duration of human missions. However, planned long-term missions carry the danger of astronauts developing severe symptoms stemming from latent viral infections. This is especially dangerous due to the limited resources available in space missions to isolate and treat the affected individuals.

### 5.2. Protecting astronauts from virus infections

In addition to infection by reactivation of latent viruses, there is also a possibility of virus infection on space missions that increases the risk of outbreaks in modules and habitats. Due to the limited possibility of identifying the cause of infection in space, it is challenging to recognize viral infections in addition to treating them. So, how can astronauts protect themselves from viral infections? Currently, most herpesvirus infections cannot be prevented through vaccines, with the exception of VZV, the causative agent of chickenpox and zoster (Papaloukas *et al.,* 2014). A balanced diet that supports a healthy metabolism, boosting the immune response, like probiotics or foods rich in vitamins, minerals, or amino acids could in theory support the fitness of the immune system, though the research in this area is still ongoing (Perdigon *et al.,* 1995; Mora *et al.,* 2008; Crucian *et al.,* 2018). Physical exercise has been found to significantly contribute to the reduced reactivation of viruses in astronauts on the ISS (Agha *et al.,* 2020). A more drastic approach would consist of using immunostimulant drugs such as bacille Calmette–Guérin (BCG), levamisole, isoprinosine, or others (Bascones-Martinez *et al.,* 2014). Anti-herpes products like acyclovir can be used to treat herpesviruses, but these have shown toxicity with prolonged use (WHO, 2013). Current journeys to space are limited in time, but in the case of longer journeys to Mars or further, latent viruses could have a greater impact.

Currently, the standard safety procedure of human spaceflight is the preflight astronauts' quarantine and disinfection of cabins and equipment. However, we must consider the risk that some viral infections might go unnoticed during the quarantine period and cause significant harm once in the space station. Development of optimized methods for virus detection and calculating the impact of the space environment on virus spread will help address this problem and provide the basis for the development of improved protocols to control eventual outbreaks in space.

## 6. Conclusion and Outlook

Viruses are a diverse biological group that is part of microbial communities in human-inhabited space modules. As such, they can influence astronauts' well-being and may pose a health threat to the crew. Intensive research is required in the field of space virology to improve the current knowledge on the dynamics caused by space stress. The effect of extreme *g*-forces or microgravity on viruses is underrepresented. It would be, for instance, interesting to test the influence of microgravity on viral spread throughout the body. Also, studies showed that hypergravity encourages the proliferation of healthy cells (Ciofani *et al.,* 2012; Genchi *et al.,* 2016), while the effect on viruses or infected and immune cells is not known. Research of viral UV-stability could be used to develop postflight UV-based sterilization of spacecraft cabins as an easy and efficient method for viral elimination. It would be especially interesting to further investigate the inactivation of human pathogenic viruses by UV radiation in various levels of RH. Developing new antimicrobial materials is another promising method for limiting viral spread during space travel.

Since the plans of future space missions tend to be more prolonged, preflight isolation and a healthy immune system might not be enough to protect astronauts against some viruses due to the overwhelming conditions during space travel. Hence, developing new methods for the detection and treatment of viral infections in space is a relevant topic.

## Abbreviations Used

AH: absolute humidity
B cells: bursa cells, a type of lymphocyte
EBV: Epstein–Barr virus
FITC-dextran: fluorescein isothiocyanate-dextran
HEPA: high-efficiency particulate air
HHV: human herpesvirus
HSV: herpes simplex virus
ISS: International Space Station
KSHV: Kaposi's sarcoma-associated herpesvirus
RH: relative humidity
ROS: reactive oxygen species
T cells: thymus cells, a type of lymphocyte
VZV: varicella zoster virus

## Notes

Associate Editor: Lewis Dartnell
